# Partially absorbed cataractous lens in the anterior chamber revealing neglected severe ocular contusion: a case report

**DOI:** 10.1186/s12886-017-0594-0

**Published:** 2017-11-06

**Authors:** Viola Andin Dohvoma, Steve Robert Ebana Mvogo, Pepin Williams Atipo-tsiba, Emilienne Epee, Paul Jean Adrien Atangana, Samuel Amvene Nko’o, Côme Ebana Mvogo

**Affiliations:** 1 Faculty of Medicine and Biomedical Sciences, University of Yaoundé I, PO Box 3851, Messa, Yaoundé, Cameroon; 2 Marien Ngouabi University of Brazzaville, Brazzaville, Congo; 3 Faculty of Medicine and Pharmaceutical Sciences, University of Douala, Douala, Cameroon

**Keywords:** Cataract, Retinal detachment, Ocular contusion, Case report

## Abstract

**Background:**

Ocular contusion can produce severe lesions, which if not treated appropriately and promptly, can lead to visual impairment. Ocular contusion in childhood may not be reported by children.

**Case presentation:**

A 27 year old female presented with a partially absorbed cataractous lens that was dislocated into the anterior chamber of her left eye. There was mild anterior chamber reaction. She reported no history of ocular trauma; but associated findings and further investigations were in favour of a post-traumatic aetiology.

**Conclusion:**

All ocular injuries require a detailed ophthalmological examination to assess vision and the extent of lesions.

## Background

Ocular trauma is a major cause of ocular morbidity. Worldwide, approximately 1.6 million people are bilaterally blind, 2.3 million are bilaterally visually impaired and almost 19 million have unilateral blindness or visual impairment due to trauma [1]. Eye injuries are a leading cause of non-congenital unilateral blindness in children [2].

Aetiologically, such injuries are mostly accidental in children, unlike intentional violent assault in adults. Such accidental injuries, if not reported by the child, can go unnoticed by the parents, making it difficult to obtain the critical history associated with it.

## Case presentation

A 27 year old lady was seen for mild pain in her left eye which has been blind since late childhood. She noticed a whitish lesion in the left eye 2 weeks prior to hospital visit. Over the past year, she had noticed a few intermittent episodes of redness in the eye. No recent or past history of ocular trauma was reported. This was her first ever eye consultation.

**Fig. 1:**
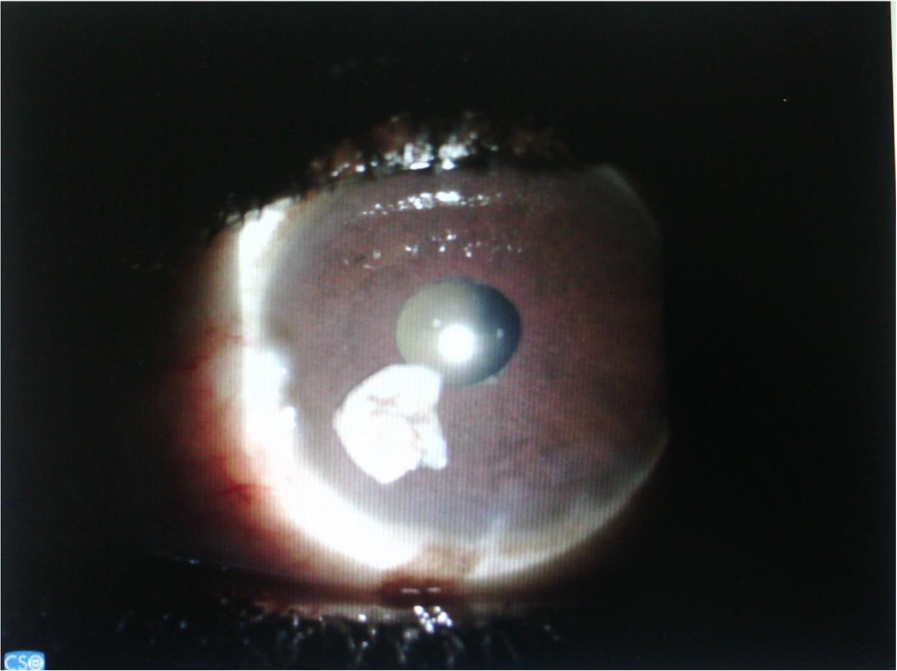
slit lamp photograph - partially absorbed cataract dislocated into the anterior chamber

Vision was normal in the right eye and no perception of light in the left eye. Anterior segment examination was normal in the right eye. There was a partially absorbed lens in the anterior chamber of the left eye (Fig. 1) and a mild anterior chamber reaction with 1+ cells. Intraocular pressures were 12 mmHg and 10 mmHg for the right and left eyes respectively. Extraocular movements were full in both eyes. Pupillary light reflex was normal in the right eye and absent in the left eye.

**Fig. 2:**
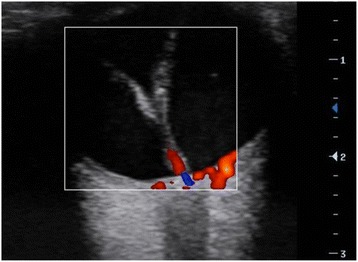
ocular ultrasound - a V-shaped membrane attached to the optic nerve head, indicative of a total retinal detachment

**Fig. 3:**
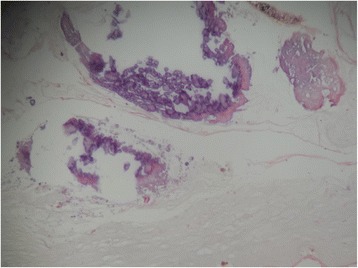
histology of lens material (stained with haematoxylin and eosin seen at × 40 magnification) - fibrous calcified tissue

A yellowish glow was observed from the retina of the left eye (Fig. 1). An old complete retinal detachment and a large retinal tear at the equator between the 2 O’clock and 3 O’clock meridians were observed on three-mirror contact lens examination. Retinal examination was normal for the right eye. On grey-scale ultrasound, the detached retina was seen as an echogenic V-shaped membrane extending from the optic disc into the anterior part of the vitreous (Fig. 2). Fine hyper-echogenic particles were present in the vitreous, indicative of vitreous haemorrhage. Histology of the extracted material from the anterior chamber revealed an amorphous fibrous calcified tissue (Fig. 3).

We arrived at a diagnosis of neglected ocular contusion, which could explain all the findings in this patient. The lens material in the anterior chamber was extracted through a 3.2 mm corneo-scleral tunnel incision. Topical antibiotics and steroids were used in the post-operative period. The pain resolved completely thereafter and the eye has remained quiet at her last follow-up visit 1 year after surgery (Table 1).

**Table 1:** Timeline

1	Intermittent redness in left eye over the past year
2	Whitish lesion in her left eye
3	Mild pain in her left eye
4	Examination reveals dislocated lens material in the anterior chamber with anterior chamber reaction; retinal hole and old retinal detachment.
5	Surgical extraction of lens material
6	Resolution of pain

## Discussion and conclusions

Ocular contusion can cause damages in both the anterior and posterior segments. The anterior segment is affected by the coup and compression mechanisms. The contre-coup mechanism accounts for posterior segment injuries as the shock waves that cross the eye strike the posterior pole.

Cataract in ocular contusion results either from a dysfunction of the lens epithelium, leading to oedema of superficial cortical lens fibres that subsequently undergo degeneration [3] or from a rupture of the anterior lens capsule. Bannit et al. in 2009 reported 3 cases of blunt trauma causing rupture of the anterior lens capsule with cataract formation [4]. They proposed that the anterior lens capsule may have been torn by direct contusion from rapid focal indentation of the cornea onto the lens (coup injury) or by a fluid-mechanical, anteriorly directed rebound of the vitreous, bursting open the anterior capsule (contre-coup injury). Posterior capsule rupture may also occur secondary to blunt trauma and lead to progressive cataract formation [5, 6]. Partially exposed lens cortex can cause little or no anterior chamber reaction. In our patient, there was mild anterior chamber reaction, indicative of the presence of some cortical material. Elsewhere, spontaneous cataract absorption has been reported with no secondary uveitis nor glaucoma [7, 8].

Equatorial stretching from ocular compression can disrupt the lens capsule, the ciliary zonule, or both. Partial or total zonular damage may occur in blunt injuries, resulting in partial or total dislocation of the lens [9]. Zonular damage probably occurred in our patient, leading to secondary dislocation. Primary post traumatic lens dislocation into the anterior chamber is usually associated with pain due to angle-closure glaucoma.

Posterior segment injuries which result from the contre-coup mechanism may include commotio retinae, macular oedema and holes, retinal tears and vitreous haemorrhages. Traumatic breaks or retinal detachment may not occur immediately after injury. Goffstein and Burton observed that 60% were diagnosed within 8 months of injury [10]. Immediate retinal detachment results from impact necrosis, while late detachment results from leakage of fluid from the choroid into the sub-retinal space and movement of vitreous fluid through retinal breaks [11].

Ocular contusion may not be reported by children or adolescents, yet be responsible for severe lesions which could be sight-threatening. Parents, guardians and child care givers should pay attention to any observed changes in the eye or visual behaviour and seek immediate ophthalmic care.
